# Enhanced Degradation of Petroleum and Chlorinated Hydrocarbons by a Dual-Bacteria System

**DOI:** 10.3390/toxics14020119

**Published:** 2026-01-27

**Authors:** Haochen Zhang, Yibin Yang, Haishan Qi, Juncheng Liu, Xiaoqiang Jia

**Affiliations:** 1 Department of Biochemical Engineering, School of Synthetic Biology and Biomanufacturing, Tianjin University, Tianjin 300350, China; 2 State Key Laboratory of Synthetic Biology, Tianjin University, Tianjin 300350, China; 3 Frontiers Science Center for Synthetic Biology (Ministry of Education), Tianjin University, Tianjin 300350, China; 4 Tianjin Huakan Environmental Protection Technology Co., Ltd., Tianjin 300000, China

**Keywords:** biodegradation, organic pollutants, petroleum hydrocarbons, aromatic hydrocarbons, chlorinated hydrocarbons

## Abstract

In this study, the gradient pressure enrichment method was first used to screen out an environmental bacterium with the degradation ability of typical petroleum hydrocarbons such as phenanthrene and n-hexadecane, identified as *Pseudomonas* and named TB-1, from soil samples collected from 9 crude oil-contaminated sites; then, enhanced degradation of mixed organic pollutants, including petroleum and chlorinated hydrocarbons which are commonly coexistent, was achieved by a dual-bacteria system, with the addition of a laboratory storage strain *Pseudomonas* BL5. The degradation rate of phenanthrene and n-hexadecane by the dual-bacteria system was lower compared with the single bacterium *Pseudomonas* TB-1 under the tested conditions: phenanthrene degradation decreased from 44.2% to 23.1%, and n-hexadecane degradation decreased from 77.9% to 54.7% at a pollutant concentration of 100 mg/L after 7 days of cultivation. In contrast, the degradation ability of the dual-bacteria system against the mixed pollutants composed of petroleum and chlorinated hydrocarbons was good, with a degradation rate of 82.2% for phenanthrene, 89.2% for n-hexadecane, 73.1% for p-chlorobenzene, and 95.7% for dichloroethane with each concentration of 100 mg/L after 7 days. These results indicate that, although the dual-bacteria system does not enhance degradation under single-hydrocarbon conditions, its performance under chemically complex co-contamination suggests a potential cooperative or complementary interaction between the two strains. Such interactions are proposed here as a working hypothesis rather than a confirmed mechanism. Overall, the defined dual-*Pseudomonas* system shows promising potential for the treatment of environments co-contaminated with petroleum and chlorinated hydrocarbons.

## 1. Introduction

Petroleum hydrocarbons and chlorinated hydrocarbons, which are commonly coexistent, are pervasive environmental contaminants that pose substantial ecological and human-health risks. Petroleum contamination—originating from oil exploration, transportation, refining and accidental spills [1]—consists of complex mixtures of aliphatic, aromatic, and polycyclic aromatic hydrocarbons (PAHs), many of which are persistent, bioaccumulative and carcinogenic [2,3]. Chlorinated hydrocarbons (e.g., chlorobenzenes, dichloroethane) originate predominantly from industrial solvents, degreasing operations and chemical manufacturing [4]; many exhibit high toxicity, mobility in groundwater and resistance to natural attenuation [5], thereby representing persistent threats to drinking-water safety and ecosystem health [6]. These properties make both pollutant classes high-priority targets for remediation efforts worldwide.

Microbial bioremediation offers an environmentally compatible and cost-effective alternative to intensive physicochemical treatments. Advantages include in situ applicability, low secondary pollution, and the capacity of microbial communities to perform stepwise degradation of complex mixtures via metabolic cooperation [7,8,9]. However, microbial remediation also faces limitations: degradation rates can be slow and are sensitive to environmental factors (temperature, nutrient availability, redox status); many well-characterized degraders are substrate-specific and perform poorly when confronted with chemically diverse co-contaminants; and bioaugmentation success is often hampered by poor survival or competitiveness of introduced strains in native soil microbiomes [10,11]. These constraints are particularly acute in mixed-pollutant scenarios, where single strains may lack the enzymatic repertoire or environmental robustness required to mineralize multiple contaminant classes fully [12,13].

The co-occurrence of petroleum hydrocarbons and chlorinated hydrocarbons in contaminated environments is largely attributable to their overlapping industrial sources and historical usage patterns. petroleum hydrocarbons are released extensively during crude oil extraction, petrochemical processing, storage, and accidental leakage, while chlorinated hydrocarbons (such as chlorinated solvents and chlorinated aliphatics) are widely applied as degreasing agents, extraction solvents, and intermediates in chemical manufacturing. Due to the frequent coupling of petroleum refining with solvent-based cleaning and chemical processing operations, mixed spills involving both petroleum hydrocarbons and chlorinated hydrocarbons are common in industrial zones, refinery sites, and long-term chemical storage facilities. Moreover, improper disposal of waste solvents, leakage from aging underground storage tanks, and co-discharge of oily wastewater with chlorinated organics further promote their simultaneous release into soil and groundwater. Once released, the differential volatility and solubility of petroleum hydrocarbons and chlorinated hydrocarbons enhance their spatial convergence: petroleum hydrocarbons tend to accumulate in the vadose and capillary zones, while highly mobile chlorinated hydrocarbons readily migrate downward to aquifers, resulting in vertical and lateral overlap within the subsurface.

As a result of these combined industrial and environmental transport mechanisms, petroleum hydrocarbons and chlorinated hydrocarbons frequently co-occur at contaminated sites [14] worldwide, creating complex pollutant mixtures that pose significant challenges to bioremediation. First, the two pollutant classes differ markedly in physicochemical properties and preferred biodegradation pathways: hydrophobic PAHs [15] require enhanced bioavailability [16] (e.g., biosurfactants or sorption/desorption processes) and are commonly degraded via oxygenase-mediated pathways, whereas chlorinated compounds often require dehalogenation reactions [17] that can proceed under different redox conditions (including anaerobic pathways) and may be inhibited by oxygenases’ substrate specificity. Second, toxic effects or inhibitory metabolites from one pollutant class can suppress the microbial populations responsible for degrading the other, and substrate competition or metabolic interference may further reduce overall mineralization [18]. Third, heterogeneity in soil/water matrices (sorption to organic matter, partitioning to non-aqueous phases) creates mass-transfer limitations that differ between hydrophobic hydrocarbons and more water-mobile chlorinated compounds [19,20], complicating single-technology solutions. Consequently, co-contamination typically necessitates integrated or multi-functional remediation approaches rather than single-pathway treatments.

To overcome single-strain limitations, researchers have increasingly explored defined mixed-strain systems and enriched consortia. Such assemblages can provide metabolic complementarity (different taxa executing sequential or parallel degradation steps), enhanced bioavailability via biosurfactant producers, detoxification of inhibitory intermediates, and greater ecological resilience under fluctuating environmental conditions. Empirical studies and reviews show that mixed cultures—whether enriched from contaminated sites or synthetically assembled—often outperform monocultures in degrading complex hydrocarbon mixtures [21]. Nonetheless, systematic studies specifically targeting the simultaneous biodegradation of petroleum hydrocarbons and chlorinated hydrocarbons remain comparatively scarce.

Petroleum hydrocarbon is often addressed by microbial bioremediation using natural or enriched microbial consortia rather than monocultures, because complex hydrocarbon mixtures (alkanes, PAHs) typically require different enzymatic capabilities for individual components [22]. For example, Santisi et al. used a defined consortium composed of *Alcanivorax borkumensis* SK2, *Rhodococcus erythropolis* HS4, and *Pseudomonas stutzeri* SDM isolated from natural seawater crude-oil enrichments [23], demonstrating that the mixed consortia outperformed single strains in degrading a range of n-alkanes in crude oil. Other work has shown that even among consortia constructed with multiple petroleum-degrading strains, choice of strains and their ratios matters greatly: Wu et al. systematically tested 27 consortia formed from eight bacteria (including *Brevundimonas* sp. Tibet-IX23, *Bacillus firmus* YHSA15, *Microbacterium oxydans* CV8.4, *Rhodococcus erythropolis* SBUG 2052, *Pseudomonas alcaligenes* NBRC, among others), and identified an optimal consortium combination that maximized degradation efficiency under experimental conditions [24].

More recently, beyond purely bacterial consortia, researchers have constructed fungal–bacterial consortia for petroleum hydrocarbon remediation [25]. For example, in one study on diesel/biodiesel-contaminated soil, a mixed consortium composed of *Trichoderma koningiopsis* P05R2, *Serratia marcescens* P10R19, and *Burkholderia cepacia* P05R9 achieved degradation of total petroleum hydrocarbons (C5–C40) and PAHs with removal rates exceeding those of individual strains demonstrating that metabolic complementarity (e.g., fungal hyphae facilitating bacterial dispersion, increased enzyme or biosurfactant production) can significantly improve degradation of complex mixtures [26]. However, not all consortia perform better than single strains, especially under suboptimal conditions: Nnabuife et al. using *Pseudomonas* strain W15, *Providencia vermicola* strain W8, and *Serratia marcescens* strain W13 isolated from a crude-oil contaminated site, their consortium performed worse (lower degradation percentage) than the best single strain (W15) when tested on 3% crude oil—likely due to antagonistic interactions, competitive exclusion, or instability in population dynamics in the mixed culture [27]. Thus, while mixed-strain consortia with well-chosen, complementary strains have shown promise in enhancing biodegradation of petroleum hydrocarbons, the selection of members, their ratios, environmental conditions, and the stability of the consortium over time are critical factors that can determine success or failure of the bioremediation [28]. Recent reviews reinforce this—arguing for rational design of microbial consortia (synthetic microbiomes) based on understanding of interspecies metabolic interactions and ecological compatibility [29].

Based on the above considerations, the present study aims to develop and evaluate a defined dual-bacteria system for the biodegradation of mixed petroleum and chlorinated hydrocarbon contaminants. An environmental isolated and petroleum hydrocarbon–degrading strain, *Pseudomonas* TB-1, was isolated from crude-oil-contaminated soils using a gradient pressure enrichment strategy, and subsequently combined with a laboratory-preserved strain, *Pseudomonas* BL5, to construct a cooperative dual-strain system. The degradation performance of this system toward representative petroleum hydrocarbons (phenanthrene and n-hexadecane) and commonly co-occurring chlorinated hydrocarbons (1,2-dichloroethane and chlorobenzene), both as individual substrates and as mixed pollutants, was systematically investigated. In addition, key operational parameters, including initial pH, inoculation ratio, and pollutant concentration, were optimized to further enhance degradation efficiency.

By integrating strain selection from contaminated environments with rational dual-strain assembly and process optimization, this study seeks to provide experimental evidence for the feasibility and advantages of mixed-culture bioremediation strategies in treating complex co-contaminated systems, and to offer practical insights for the design and implementation of microbial remediation approaches for petroleum-chlorinated hydrocarbon co-contaminated sites.

## 2. Materials and Methods

### 2.1. Screening and Isolation of Strains

Soil samples were collected from nine crude-oil-contaminated sites (Dahuangbao Wetland Nature Reserve, Tianjin, China) were independently subjected to gradient enrichment. Dominant hydrocarbon-degrading isolates obtained from each enrichment were selected and analyzed by 16S rRNA gene sequencing. Identical sequences were obtained for the isolates from different samples, and these isolates were therefore considered to represent the same strain and collectively designated as TB-1. To isolate petroleum hydrocarbon–degrading bacteria, gradient pressure enrichment was conducted in mineral salt medium (MSM) supplemented with crude oil (concentration: 100 mg/L–500 mg/L–1000 mg/L) as the sole carbon source. The cultures were incubated at 30 °C, with shaking at 200 rpm, for 5–7 days. Enrichment transfers were performed by inoculating 1% (*v*/*v*) of the culture into fresh MSM containing gradually increased hydrocarbon concentrations. After several enrichment cycles, aliquots of the enriched cultures were serially diluted and spread onto MSM agar plates coated with the crude oil. Distinct colonies were repeatedly streaked to obtain pure isolates.

### 2.2. Morphological Observation of Strains and Analysis of 16S rRNA Sequence

Colony morphology was observed on MSM agar plates after incubation at 30 °C for 48 h. Cellular morphology and Gram reaction were examined under optical microscopy using standard staining techniques.

Genomic DNA of the isolate was extracted using a commercial bacterial DNA extraction kit (TIANamp Genomic DNA Kit (TIANGEN Biotech, Beijing, China)) according to the manufacturer’s instructions. The 16S rRNA gene was amplified via PCR using universal primers 27F (5′-AGAGTTTGATCCTGGCTCAG-3′) and 1492R (5′-GGTTACCTTGTTACGACTT-3′). PCR products were purified and sequenced. The obtained sequences were compared with reference sequences in the NCBI GenBank database using BLAST (https://blast.ncbi.nlm.nih.gov/Blast.cgi?PROGRAM=blastn&PAGE_TYPE=BlastSearch&LINK_LOC=blasthome, 14 December 2025). Phylogenetic analysis was conducted using the neighbor-joining method in MEGA 11 to determine taxonomic affiliation.

### 2.3. Cultivation of Microorganisms

The isolated strain TB-1 and the laboratory-preserved strain *Pseudomonas* BL5 were routinely cultured in MSM medium at 30 °C with shaking at 200 rpm. For biodegradation experiments, bacterial cells were harvested during the exponential growth phase by centrifugation at 6000× *g* for 10 min, washed twice with sterile saline (0.9%, *w*/*v*), and resuspended in sterile MSM supplemented with corresponding hydrocarbons to the desired cell density (OD_600_ = 0.5).

For the construction of the dual-bacteria system, equal volumes (or specified ratios) of TB-1 and BL5 suspensions were mixed immediately before inoculation.

### 2.4. Strain Growth Determination

Microbial growth was monitored by measuring optical density at 600 nm (OD_600_) using a UV–visible spectrophotometer. Samples were withdrawn aseptically at predetermined time intervals. Intensive monitoring was carried out during the pre-culture period, and sampling was carried out every 2 h. The logarithmic growth period was adjusted to 3 h. The plateau period was extended to 6 h.

### 2.5. Strain Degradation Determination

Degradation experiments were conducted in 250 mL Erlenmeyer flasks containing 100 mL of MSM supplemented with phenanthrene, n-hexadecane, 1,2-dichloroethane, and chlorobenzene, individually or as a mixture. The initial concentration of each pollutant was 100 mg/L. In mixed-pollutant systems, the total concentration was maintained at 100 mg/L, with each of the four pollutants added in equal proportion (25 mg/L each). For concentration-optimization experiments, the total concentration was varied at 50, 100, 150, and 200 mg/L, with the four pollutants still added in equal proportions. Flasks were inoculated with either single-strain TB-1 or the dual-bacteria system and incubated at 30 °C, with shaking at 200 rpm for 7 days. Uninoculated flasks served as abiotic controls.

At the end of incubation, residual pollutants were extracted using organic solvents (e.g., n-hexane or dichloromethane, 1:1 *v*/*v*) and analyzed quantitatively by gas chromatography (GC). The concentrations of target organic pollutants were determined using a GC-430 gas chromatograph equipped with a flame ionization detector (FID). A HP-5 column (30 m × 0.32 mm × 0.25 μm) was used for separation. For each analysis, 1 μL of the extracted sample was injected into the GC inlet, which was maintained at 270 °C. Nitrogen was used as the carrier gas at a flow rate of 1 mL/min. The oven temperature program was set as follows: initially held at 80 °C for 1 min, then ramped to 250 °C at 15 °C/min, followed by a further increase to 280 °C at 50 °C/min, and finally maintained at 280 °C for 2 min. *Degradation efficiency* (%) was calculated as:

(1) $$Degradation\;efficiency\;\left(\%\right)=\frac{{\left(\frac{A_{s}}{A_{IS}}\right)}_{C}-\;{\left(\frac{A_{s}}{A_{IS}}\right)}_{T}}{{\left(\frac{A_{s}}{A_{IS}}\right)}_{C}}\times \;100$$

where $A_{s}$ represents the GC peak area of the target pollutant, $A_{IS}$ represents the peak area of the internal standard, ${\left(\frac{A_{s}}{A_{IS}}\right)}_{C}$ represents the normalized peak area ratio in the control group, and ${\left(\frac{A_{s}}{A_{IS}}\right)}_{T}$ represents the normalized peak area ratio in the treatment group after incubation.

### 2.6. Graphical Summary Generation

Graphical summaries are drawn by Figdraw.

## 3. Results and Discussion

### 3.1. Screening and Identification of Strains

#### 3.1.1. Morphological Observation of Strains

**Figure 1:**
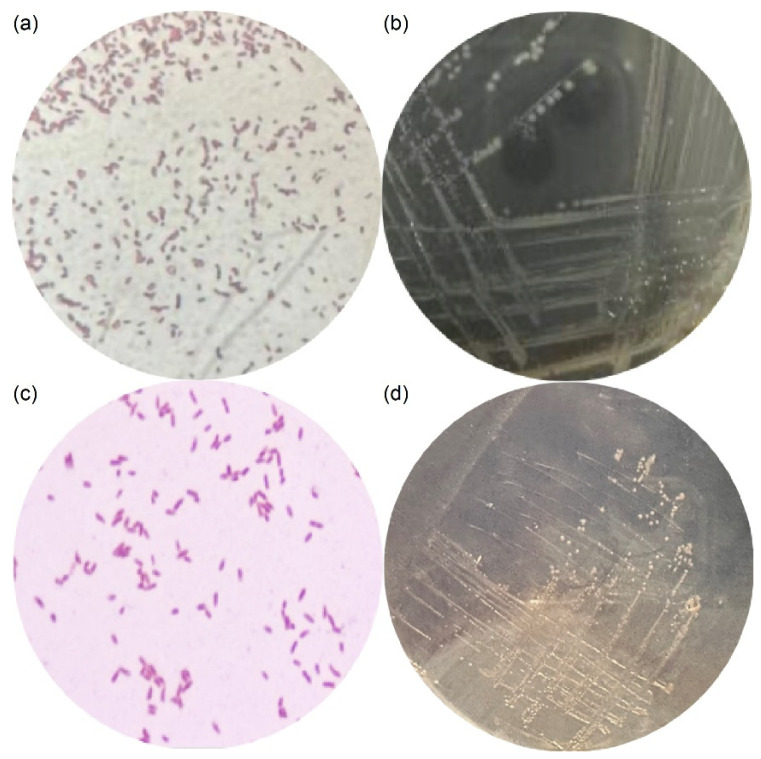
Morphological characteristics of *Pseudomonas* TB-1 and *Pseudomonas* BL5. (**a**) Gram-staining microscopic image of strain TB-1. (**b**) Gram-staining microscopic image of strain BL5. (**c**) Colony morphology of strain TB-1 on MSM agar plates. (**d**) Colony morphology of strain BL5 on MSM agar plates. MSM agar plates were supplemented with crude oil as the carbon source for TB-1 and with benzene as the carbon source for BL5.

Microscopic observation after Gram staining revealed that both strains TB-1 and BL5 were Gram-negative bacteria with a typical rod-shaped morphology (Figure 1a,c). No spore formation was observed under the tested conditions. Plate cultivation further revealed distinct colony morphologies between the two strains: TB-1 colonies appeared smaller and slightly translucent with smoother surfaces, whereas BL5 formed relatively smooth, opaque colonies with regular margins (Figure 1b,d).

These morphological characteristics are consistent with previously reported features of *Pseudomonas* sp. isolated from hydrocarbon-contaminated environments [30,31], and provide preliminary phenotypic evidence supporting the subsequent molecular identification.

#### 3.1.2. Sequence Analysis and Phylogenetic Tree Construction

**Figure 2:**
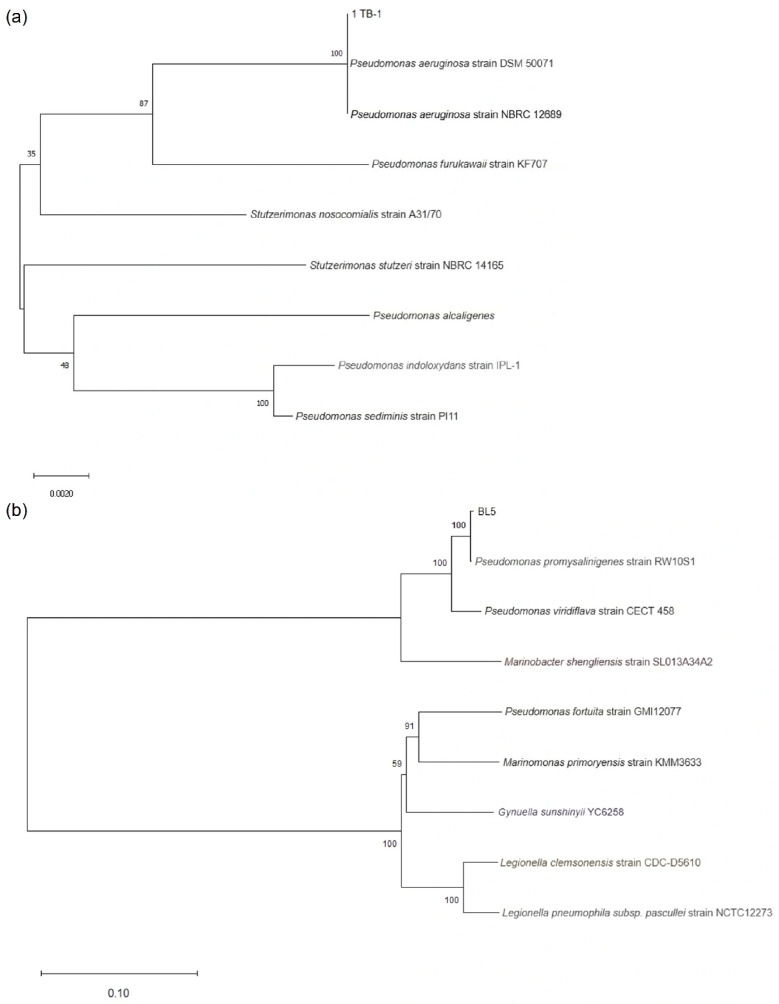
Phylogenetic trees based on 16S rRNA gene sequences showing the taxonomic positions of strains TB-1 and BL5. (**a**) Phylogenetic tree of strain TB-1. (**b**) Phylogenetic tree of strain BL5. Bootstrap values (>50%) based on 1000 replicates are shown at branch points.

Phylogenetic analysis based on 16S rRNA gene sequences showed that strain TB-1 clustered closely with reference strains of *Pseudomonas*, while BL5 grouped within the *Pseudomonas* clade (Figure 2a,b).

Both strains exhibited high sequence similarity to their closest relatives, confirming their taxonomic affiliation at the species level. The prevalence and functional diversity of *Pseudomonas* in petroleum-impacted sites are well documented and support the ecological relevance of these isolates [32,33].

The 16S rRNA-based phylogenetic assignment places both isolates within the genus *Pseudomonas*, a taxon that is well documented for its metabolic versatility and frequent involvement in hydrocarbon biodegradation [34]. *Pseudomonas* sp. have repeatedly been reported to mineralize both aliphatic n-alkanes and polycyclic aromatic hydrocarbons (PAHs) under aerobic conditions, often with high efficiencies in laboratory studies [35,36].

#### 3.1.3. Strain Growth

**Figure 3:**
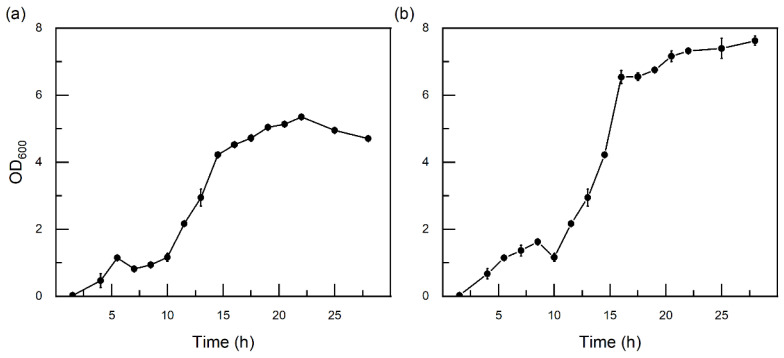
Growth curve of strain TB-1 (**a**) or BL5 (**b**) cultivated in MSM liquid medium. Cell growth was monitored by measuring optical density at 600 nm (OD_600_) at regular time intervals. All experiments were performed in triplicate (n = 3), and data are presented as mean ± standard deviation (SD). Error bars represent SD. MSM were supplemented with crude oil as the carbon source for TB-1 and with benzene as the carbon source for BL5. OD_600_ values above the linear measurement range were obtained by dilution and back-calculation and are presented for comparative purposes only.

As shown in Figure 3, both strain TB-1 and BL5 [37] exhibited a typical bacterial growth curve, with a short lag phase, a pronounced exponential growth phase, and a stationary phase over 30 h of incubation.

Rapid biomass accumulation during the exponential phase indicated good adaptability of TB-1 or BL5 to the cultivation medium and experimental conditions. Robust growth under laboratory conditions is commonly considered a prerequisite for effective biodegradation performance in bench-scale assays [38].

During cultivation in shaking flasks, a noticeable darkening of the medium is observed in some experiments, which was not due to contamination but likely some intermediate metabolites produced during cell growth and hydrocarbon utilization. As reported in the literature [39], *Pseudomonas* spp. are capable of synthesizing pigments (e.g., phenazine pigments such as pyocyanin), which can lead to visible medium color changes. For example, *Pseudomonas* aeruginosa produced greenish-blue phenazine pigments.

In addition, the production of biosurfactants and other extracellular compounds during hydrocarbon metabolism may also contribute to increased turbidity and color changes, which in turn can affect optical density measurements.

### 3.2. Degradation of Petroleum Hydrocarbons

#### 3.2.1. Degradation of Petroleum Hydrocarbons by TB-1

**Figure 4:**
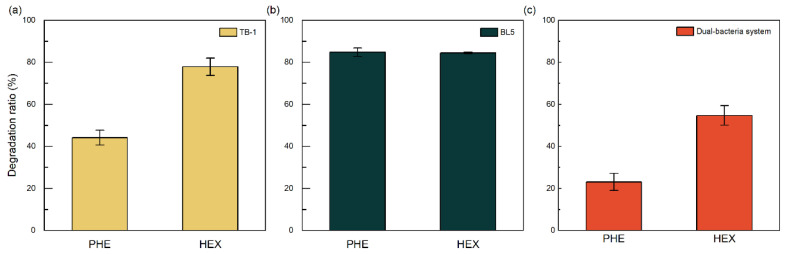
Degradation efficiencies of petroleum hydrocarbons by strain TB-1 (**a**), BL5 (**b**), dual-bacteria system (**c**) after 7 days of incubation. Phenanthrene or *n*-hexadecane was supplied individually as the sole carbon source at an initial concentration of 100 mg/L. The dual-bacteria system is composed of strains TB-1 and BL5 at a 1:1 inoculation ratio. All experiments were performed in triplicate (n = 3), and data are presented as mean ± standard deviation (SD). Error bars represent SD. Abiotic controls were included for all treatments, and their corresponding values were used to calculate degradation efficiencies, as described in Section 2.

Strain TB-1 demonstrated the ability to degrade phenanthrene or *n*-hexadecane as the sole carbon source (Figure 4a). After 7 days of incubation, the degradation efficiencies of phenanthrene and *n*-hexadecane reached 44.16% and 77.89%, respectively.

The capacity of *Pseudomonas* strains to metabolize both aromatic (PAHs) and aliphatic hydrocarbons has been widely reported, attributable to their diverse oxygenase systems and hydrocarbon-utilizing pathways [40,41].

#### 3.2.2. Degradation of Petroleum by Additional Strains BL5

Compared to the TB-1 strain, the BL5 strain exhibits differences in its degradation spectrum for petroleum hydrocarbons. As shown in Figure 4b, BL5 demonstrated higher degradation efficiencies for phenanthrene and n-hexadecane than TB-1 (84.82% and 84.48%, respectively). It was anticipated that incorporating BL5 would significantly enhance the petroleum hydrocarbon degradation efficiency of the dual-bacteria system compared to a single strain. However, subsequent experiments demonstrated that this was not the case.

#### 3.2.3. Degradation of Petroleum by Mixed Bacteria

When the TB-1 and BL5 strains were combined at a 1:1 inoculation ratio, the constructed dual-bacteria system exhibited lower degradation efficiencies for phenanthrene and n-hexadecane compared to the BL5 single-strain system (Figure 4c). At an initial concentration of 100 mg/L, the degradation efficiency for phenanthrene decreased from 44.16% to 23.09%, while that for n-hexadecane dropped from 77.89% to 54.73%.

This unexpected decrease in single-substrate degradation by the two-strain consortium may be explained by competitive interactions for a single limiting carbon source and by antagonistic effects between strains [42,43]. Additional causes may include accumulation of inhibitory metabolic intermediates and inoculum-ratio dependence, both of which have been shown to reduce consortium performance under certain conditions [44,45].

### 3.3. Degradation of Mixed Petroleum and Chlorinated Hydrocarbons by the Dual-Bacteria System

#### 3.3.1. Degradation of Mixed Pollutants by the Dual-Bacteria System

**Figure 5:**
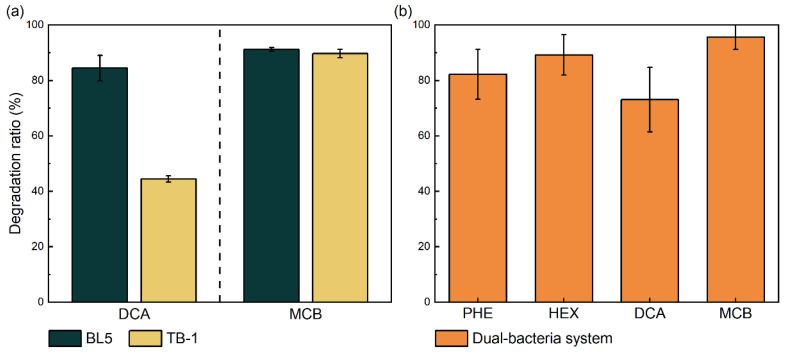
Degradation performance of single bacterial strains against chlorinated hydrocarbons and dual bacterial systems (TB-1 and BL5 mixed 1:1) against mixed pollutants (after 7 days of cultivation). (**a**) Degradation efficiency of TB-1 and BL5 for chlorinated hydrocarbons (1,2-dichloroethane or chlorobenzene). (**b**) Degradation efficiency of the dual-bacteria system for mixed pollutants consisting of 100 mg/L mixed pollutants (phenanthrene, *n*-hexadecane, 1,2-dichloroethane, and chlorobenzene mixed by equal mass). All experiments were performed in triplicate (n = 3), and data are presented as mean ± standard deviation (SD). Error bars represent SD. Abiotic controls were included for all treatments, and their corresponding values were used to calculate degradation efficiencies, as described in Section 2.

Given the favorable degradation of chlorinated hydrocarbons by the single-bacterial system (Figure 5a) and considering that the reduced degradation rate of petroleum hydrocarbons in the dual-bacterial system mentioned in Section 3.2.3 is associated with resource competition and antagonistic effects, substrate preference among strains can reflect differences in catabolic gene content and regulation, and such functional complementarity is often exploited in consortium design [41]. Therefore, we attempted to incorporate additional pollutants to mitigate this effect.

As anticipated, the dual-bacteria system exhibited effective degradation of chlorinated hydrocarbons, including 1,2-dichloroethane and chlorobenzene, as well as petroleum hydrocarbons under mixed-pollutant conditions (Figure 5b). When four pollutants were present at equal initial concentrations (100 mg/L each), the system maintained high removal efficiencies for all components (ranging from 73.1% to 95.7%). Simultaneous removal of petroleum hydrocarbons and chlorinated organics is challenging because the two classes often follow distinct degradation pathways (e.g., oxygenase-mediated oxidation vs. reductive or hydrolytic dehalogenation); nonetheless, integrated microbial systems have been reported to accomplish concurrent transformation when complementary functions coexist within the community [12,13,14].

Such performance enhancement by defined consortia has been frequently observed and is commonly attributed to metabolic complementarity, cross-feeding, biosurfactant production, or detoxification of inhibitory intermediates [9,10,11].

#### 3.3.2. Optimize the Degradation of Mixed Pollutants by the Dual-Bacteria System

**Figure 6:**
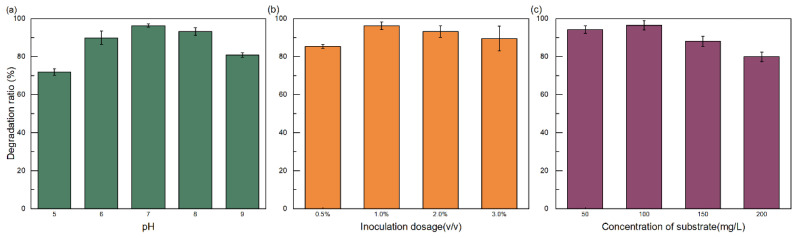
Effects of environmental and operational parameters on the degradation of mixed pollutants by the dual-bacteria system. (**a**) Effect of pH (5–9). (**b**) Effect of inoculation ratio (0.5–3%). (**c**) Effect of initial pollutant concentration (50–200 mg/L). Degradation efficiencies were determined after 7 days of incubation. All experiments were performed in triplicate (n = 3), and data are presented as mean ± standard deviation (SD). Error bars represent SD. Abiotic controls were included for all treatments, and their corresponding values were used to calculate degradation efficiencies, as described in Section 2.

Environmental parameters, including pH, inoculation ratio, and initial pollutant concentration, significantly influenced the degradation performance of the dual-bacteria system (Figure 6). Optimal degradation was achieved under near-neutral pH conditions (pH = 7), moderate inoculation ratios (1%), and intermediate pollutant concentrations (100 mg/L). The sensitivity of biodegradation to such operational factors is well recognized, and optimization of these parameters is a standard approach to maximize removal in laboratory and field trials [3,15,16]. Together, the observed robustness across a range of conditions supports the applicability of the TB-1/BL5 consortium under varied environmental contexts.

### 3.4. Limitations

It should be noted that the biodegradation experiments in this study were conducted in liquid mineral medium, which generally allows faster microbial degradation compared to soil or groundwater systems due to better mass transfer and pollutant availability. Therefore, caution should be taken when extrapolating these results to real environmental conditions, and further studies in soil or groundwater systems are required to confirm the effectiveness of the dual-strain consortium in situ.

Another limitation is that the long-term population dynamics of the dual-strain consortium remain unknown. Although the mixed culture showed stable degradation over the short-term experiments, environmental factors in soil or groundwater could affect the survival and activity of the strains. Further studies are needed to evaluate the sustainability and feasibility of maintaining the consortium under realistic environmental conditions.

Another limitation is that under controlled laboratory conditions, the biodegradation efficiency of the tested strains did not vary markedly within the investigated ranges of pH (5–9), inoculum size (0.5–3%, *v*/*v*), and pollutant concentration (50–200 mg/L). Similar observations have been reported in previous studies, where hydrocarbon-degrading bacteria or consortia maintained substantial degradation activity across moderate variations of key physicochemical parameters. For example, a consortium containing *Pseudomonas* and Rhodococcus strains showed sustained crude oil degradation across a pH range of 6–9, with only moderate decreases at more extreme values, indicating that biodegradation performance is relatively robust within typical experimental ranges [4]. Likewise, phenol-degrading *Pseudomonas* strains have been reported to retain degradation capability across different inoculum sizes, although degradation rates may vary [5]. Moreover, *Pseudomonas* spp. are widely recognized for their metabolic versatility and biosurfactant production, which can enhance hydrocarbon bioavailability and contribute to stable degradation performance under variable laboratory conditions [6].

It should be emphasized that these results reflect controlled laboratory conditions, in which environmental parameters fluctuate within limited and defined ranges. In contrast, real contaminated environments are characterized by much broader and more dynamic variations in physicochemical conditions. Therefore, during strain screening and performance evaluation, it is essential to consider not only degradation efficiency under optimal conditions but also the stability and adaptability of the strains under fluctuating conditions.

## 4. Conclusions

In this study, a bacterial strain with representative petroleum hydrocarbon-degrading capability was screened from soil samples collected at nine crude-oil-contaminated sites using a gradient pressure enrichment approach and was identified as *Pseudomonas* TB-1. Subsequently, this strain was combined with a laboratory-preserved strain, *Pseudomonas* BL5, to construct a dual-bacteria system, and its degradation performance toward typical petroleum hydrocarbons (phenanthrene and n-hexadecane) and commonly detected chlorinated hydrocarbons (1,2-dichloroethane and chlorobenzene) in mixed-pollutant systems was systematically evaluated.

The improved degradation observed in the dual-bacteria system may be due to synergistic interactions, such as metabolic complementarity and increased tolerance to toxic compounds, which could play an important role in the treatment of complex co-contaminated systems. In addition, optimization of environmental factors and inoculation ratios further enhanced pollutant removal, demonstrating the robustness and adaptability of the constructed dual-bacteria system.

Overall, this study provides experimental evidence supporting the applicability of *Pseudomonas*-based dual-bacteria systems for the remediation of environments co-contaminated with petroleum hydrocarbons and chlorinated hydrocarbons, and offers a feasible biological alternative for future field-scale remediation strategies.

## Data Availability

The original contributions presented in this study are included in the article. Further inquiries can be directed to the corresponding authors.

## Abbreviations

The following abbreviations are used in this manuscript:

PAHs	Polycyclic aromatic hydrocarbons
MSM	Mineral salt medium
PHE	phenanthrene
HEX	Hexadecane
DCA	1,2-Dichloroethane
MCB	Chlorobenzene
